# Regulation of airway remodeling in asthma: The synergistic effects of nanotechnology and Hippo pathway activation

**DOI:** 10.1063/5.0251258

**Published:** 2025-07-18

**Authors:** Zhihua Li, Mingrui Yan, Jiaqi Bi, Biwen Mo

**Affiliations:** 1 Postdoctoral Innovation Practice Base, The Second Affiliated Hospital of Guilin Medical University, Guilin 541199, Guangxi, People's Republic of China; 2 Department of Respiratory Medicine, The First Affiliated Hospital of Guangxi Medical University, Nanning 530021, Guangxi, People's Republic of China; 3 College of Pharmacy, Guilin Medical University, Guilin 541199, Guangxi, People's Republic of China

## Abstract

Asthma is a prevalent chronic respiratory condition characterized by airway remodeling, which contributes to irreversible airway narrowing and impaired lung function. Current therapies fail to effectively address this structural alteration. This study introduces an innovative strategy combining Quercetin, a natural flavonoid with anti-inflammatory and anti-proliferative properties, and chitosan-alginate nanoparticles (CA-NPs) to enhance bioavailability and therapeutic efficacy. By activating the Hippo signaling pathway, CA-NPs-Quercetin inhibits the proliferation and migration of human airway smooth muscle cells (HASMCs), key drivers of airway remodeling. *In vitro* experiments demonstrated significant suppression of HASMC proliferation and migration, with increased apoptosis rates attributed to the phosphorylation of YAP and TAZ, which restricted their nuclear translocation. *In vivo* experiments showed that CA-NPs-Quercetin significantly improved airway inflammation in asthmatic mice, reducing the total number of inflammatory cells and eosinophils in the bronchoalveolar lavage fluid, with a significant reduction in IL-4 expression (56.65%) and significant upregulation of IL-10 (130.91%) and IFN-γ (156.58%) levels. Moreover, CA-NPs-Quercetin reduced collagen deposition in lung tissue (Quercetin group reduced by 42.53% and CA-NPs-Quercetin group reduced by 73.20%) and improved lung function. This study provides a new approach for the application of Quercetin in asthma treatment and reveals the potential regulatory role of the Hippo pathway in asthma-related airway remodeling, demonstrating its broad application prospects in the treatment of respiratory diseases.

## INTRODUCTION

Asthma is a common chronic respiratory disease affecting the health of hundreds of millions of people worldwide.^1^ While current medications are effective in alleviating acute asthma symptoms, patients often continue to experience airway remodeling during long-term treatment.^2–4^ Airway remodeling is characterized by thickening of the airway walls, proliferation of smooth muscle cells, fibrosis of the extracellular matrix, and angiogenesis. These changes exacerbate airway hyperreactivity (AHR), leading to irreversible airway narrowing and a decline in lung function.^5^ Despite extensive research into the pathogenesis of asthma and its clinical interventions, effectively inhibiting airway remodeling remains a significant challenge in asthma management.^6^

Airway remodeling is primarily driven by the abnormal proliferation and migration of airway smooth muscle cells.^7^ In the airways of asthma patients, the number of airway smooth muscle cells significantly increases, and their enhanced migratory capacity further exacerbates airway narrowing.^8^ In addition to the proliferation of smooth muscle cells, the deposition of matrix proteins and inflammatory responses in the airways also contribute to structural changes.^9^ Numerous studies have shown that airway smooth muscle cells are not only executors of airway contraction in asthma but also play a central role in chronic inflammation and airway remodeling. Therefore, finding effective strategies to inhibit the abnormal behavior of airway smooth muscle cells is of critical clinical importance in preventing asthmatic airway remodeling.

In recent years, the Hippo signaling pathway has gained increasing attention as an emerging regulatory mechanism of cell proliferation and migration.^10^ The Hippo pathway promotes the phosphorylation of YAP (Yes-associated protein) and TAZ (transcriptional coactivator with PDZ-binding motif), preventing their entry into the nucleus and thereby inhibiting the expression of cell proliferation-related genes.^11^ In various tissues and organs, the Hippo pathway is regarded as a crucial regulator of cell homeostasis and tissue size.^12^ In airway smooth muscle cells, the activity of the Hippo pathway may play an essential role in suppressing abnormal proliferation and migration.^13,14^ However, research on the specific role of the Hippo pathway in asthmatic airway remodeling remains limited, highlighting the scientific importance of exploring its regulatory potential in this context.

The flavonoid Quercetin has demonstrated significant therapeutic potential in various diseases due to its broad anti-inflammatory, antioxidant, and anti-proliferative activities.^15,16^ Quercetin regulates cellular functions through multiple mechanisms, inhibiting the release of inflammatory factors and reducing abnormal cell proliferation.^17^ In studies of respiratory diseases, Quercetin has been shown to intervene in airway inflammation and airway remodeling through various pathways.^18,19^ However, due to its poor water solubility and low bioavailability, the stability of Quercetin *in vivo* is limited, affecting its therapeutic efficacy.^20^ To overcome this limitation, recent research has employed nanotechnology as a drug delivery system to enhance Quercetin's stability and bioactivity. A mesoporous bioactive glass (Quercetin/MBG) nanoparticle drug delivery system has been developed, which features a sustained release of Quercetin. This system effectively reduces the frequency of Quercetin injections while producing good therapeutic effects.^21^ By preparing Quercetin-loaded nanoliposomes under high-pressure homogenization (HPH) at varying pressures (up to 150 MPa) and cycles (up to 3), the potential of Quercetin-loaded liposomes against colon cancer cells was confirmed.^22^ In addition, researchers have developed a nano-formulation of flavonoid Quercetin (QU) in a self-nanoemulsifying drug delivery system (SNEDDS), which exhibited significantly higher bioavailability. Compared to standard Quercetin formulations, the nano-formulation more effectively inhibited histopathological features, necrosis, inflammatory cell infiltration, and vascular congestion in MetS hearts.^23^ Furthermore, previous studies have also used mesoporous silica nanoparticles (SBA-15) as the primary carrier material for therapeutic drug delivery, successfully loading Quercetin. The Q-SBA-15 system induced apoptosis via the cysteine protease-mediated PI3K/AKT/mTOR signaling pathway, improving cell apoptosis, and could potentially be used as an anticancer agent for lung cancer.^24^ Chitosan-alginate nanoparticles (CA-NPs), as a biocompatible nanomaterial, offer excellent drug-loading and delivery capabilities, providing new possibilities for the clinical application of Quercetin.

Based on this, the aim of this study is to develop chitosan-alginic acid nanoparticles loaded with Quercetin (CA-NPs-Quercetin) and inhibit the proliferation and migration of airway smooth muscle cells by activating the Hippo signaling pathway, thereby preventing airway remodeling in asthma. The efficacy and safety of this complex may be evaluated through both *in vitro* and *in vivo* experiments, with a detailed investigation into its effects on airway smooth muscle cell proliferation, migration, and the Hippo signaling pathway. Ultimately, this study aims to offer a new intervention strategy for asthma treatment and provide a scientific basis for the application of Quercetin in respiratory diseases. The findings are expected to open new avenues for the clinical management of asthma, particularly in preventing airway remodeling, highlighting its potential clinical value.

## RESULTS

### Successful preparation and characterization of CA-NPs-Quercetin

**FIG. 1.**
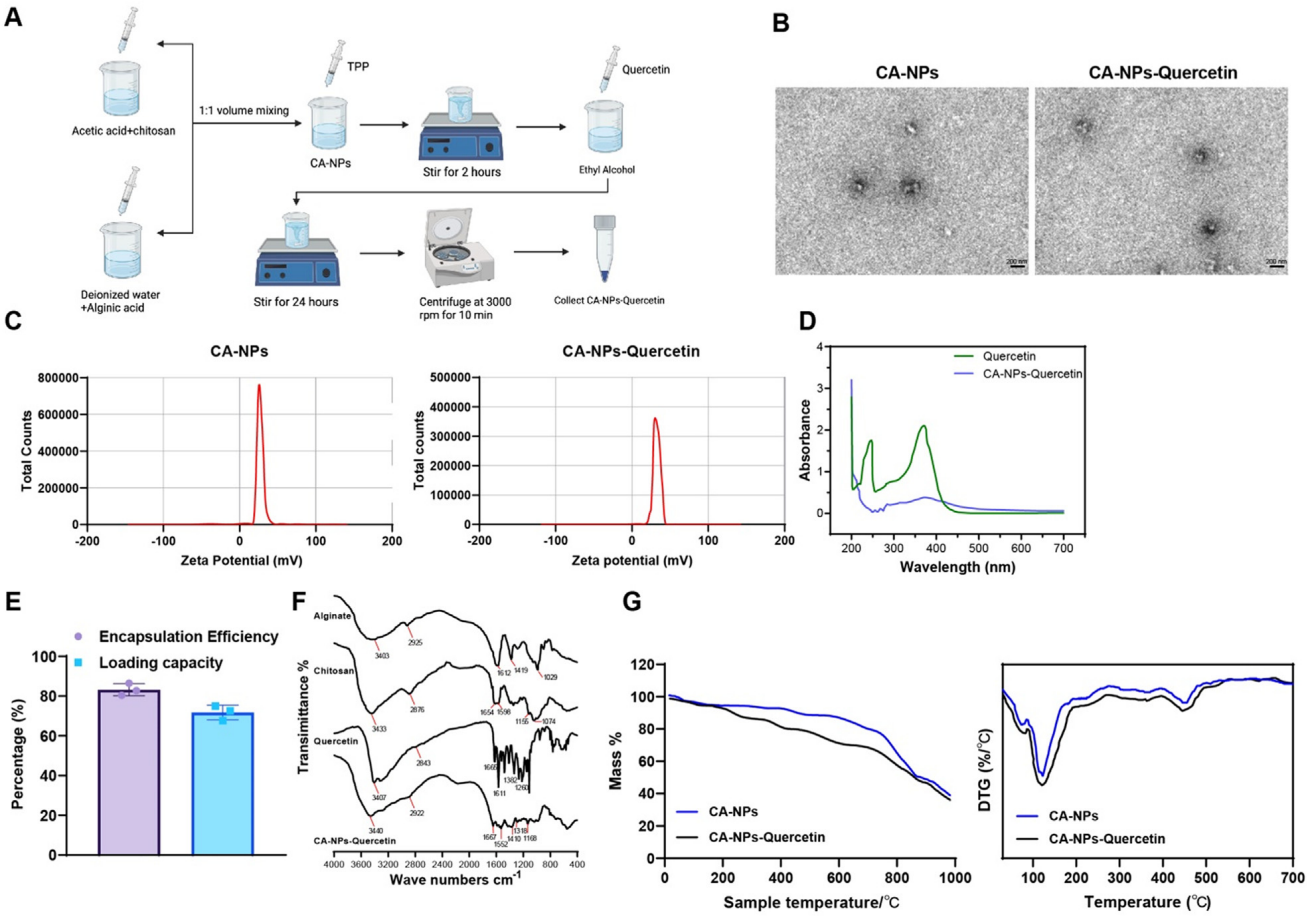
Preparation and characterization of CA-NPs and CA-NPs-Quercetin. (a) Schematic illustration of the preparation process of CA-NPs-Quercetin. (b) TEM images showing the morphology of CA-NPs and CA-NPs-Quercetin (scale bar = 200 nm). (c) Zeta potential measurement of CA-NPs and CA-NPs-Quercetin using a laser particle size analyzer. (d) Absorption spectra of free Quercetin and Quercetin-loaded nanoparticles. (e) Statistical analysis of the EE and LC of Quercetin. (f) FTIR spectra analysis of CA-NPs-Quercetin. (g) TGA and DTG analysis of evaluating the thermal stability of CA-NPs-Quercetin.

CA-NPs were successfully prepared using ionic cross-linking, and Quercetin was loaded onto their surface via physical adsorption, forming the CA-NPs-Quercetin complex [Fig. 1(a)]. Transmission electron microscopy (TEM) results showed that both CA-NPs and CA-NPs-Quercetin exhibited a regular spherical structure with good dispersibility [Fig. 1(b)].

Laser particle size analysis revealed that the Zeta potential of CA-NPs was +25 ± 3 mV, while the Zeta potential of CA-NPs-Quercetin increased slightly to +30 ± 2 mV, indicating that Quercetin loading slightly enhanced the surface charge of the nanoparticles [Fig. 1(c)]. These findings suggest that CA-NPs-Quercetin possesses excellent stability and dispersibility, making it suitable for further biomedical applications.

The encapsulation efficiency (EE) and loading capacity (LC) of Quercetin were determined using UV–visible spectrophotometry (UV–Vis) and high-performance liquid chromatography (HPLC). The absorbance of Quercetin was measured at 370 nm, and the EE of CA-NPs-Quercetin was calculated to be 84 ± 2%, with an LC of 73 ± 4% based on the standard curve [Figs. 1(d) and 1(e)]. Fourier transform infrared spectroscopy (FTIR) spectroscopy analysis revealed a significant OH stretching vibration peak at 3400 cm^−1^ in CA-NPs-Quercetin, indicating the successful incorporation of Quercetin [Fig. 1(f)]. Thermogravimetric analysis (TGA) and derivative thermogravimetry (DTG) results showed that both CA-NPs and CA-NPs-Quercetin exhibited high thermal stability in the 200–600 °C range. The thermal degradation rate of CA-NPs-Quercetin was slightly higher than that of CA-NPs in the 300–400 °C range, further confirming the Quercetin loading [Fig. 1(g)].

The drug release experiment conducted under varying pH conditions demonstrated that the release rate of Quercetin from CA-NPs-Quercetin was significantly higher under acidic conditions (pH 5.5) compared to neutral conditions (pH 7.4). At 37 °C, the cumulative release of Quercetin within 24 h was 65% at pH 5.5, whereas it was only 30% at pH 7.4 (Fig. S1A). These results indicate that CA-NPs-Quercetin exhibits favorable drug release properties in acidic environments, making it suitable for applications in inflammatory microenvironments.

Scanning electron microscopy (SEM) was used to monitor changes in morphology and particle size under different pH conditions. The results showed that the morphology and particle size of CA-NPs-Quercetin remained stable under both pH 7.4 and 5.5 conditions, with no significant changes observed (Fig. S1B). Over the 24-h observation period, the nanoparticles did not exhibit notable degradation or aggregation, suggesting that CA-NPs-Quercetin maintains good stability in both physiological and acidic environments, supporting its potential for further application studies.

### Uptake and distribution of CA-NPs-Quercetin in HASMCs and mice

To investigate the uptake and localization of CA-NPs-Quercetin in airway smooth muscle cells, CA-NPs-Quercetin was labeled with the green fluorescent lipophilic dye PKH67 (Fig. S2A). After co-incubation for 3, 6, and 12 h, the distribution of the labeled nanoparticles within the cells was observed using confocal microscopy. The results showed that CA-NPs-Quercetin was taken up by human airway smooth muscle cells (HASMCs) as early as 6 h post-treatment, with uptake continuing to increase by 12 h. The CA-NPs-Quercetin exhibited strong green fluorescent signals, primarily localized in the cytoplasmic region of the cells (Fig. S2B).

In the *in vivo* experiment, fluorescently labeled CA-NPs-Quercetin was administered via tail vein injection, and the biodistribution of the nanoparticles in mice was monitored using an *in vivo* imaging system. Imaging was performed at 3 and 6 h post-injection to track the accumulation of nanoparticles in major organs (heart, liver, kidneys, lungs, and spleen). The results showed that CA-NPs-Quercetin exhibited the strongest fluorescence signals in the liver and kidneys 3 h after injection, with the signals decreasing by 6 h, indicating that the nanoparticles were primarily metabolized and excreted through these organs (Fig. S2C).

In conclusion, CA-NPs-Quercetin demonstrated efficient uptake by cells and tissues both *in vitro* and *in vivo* and displayed favorable biodistribution characteristics *in vivo*.

### CA-NPs-Quercetin exhibits good biocompatibility and low cytotoxicity

**FIG. 2.**
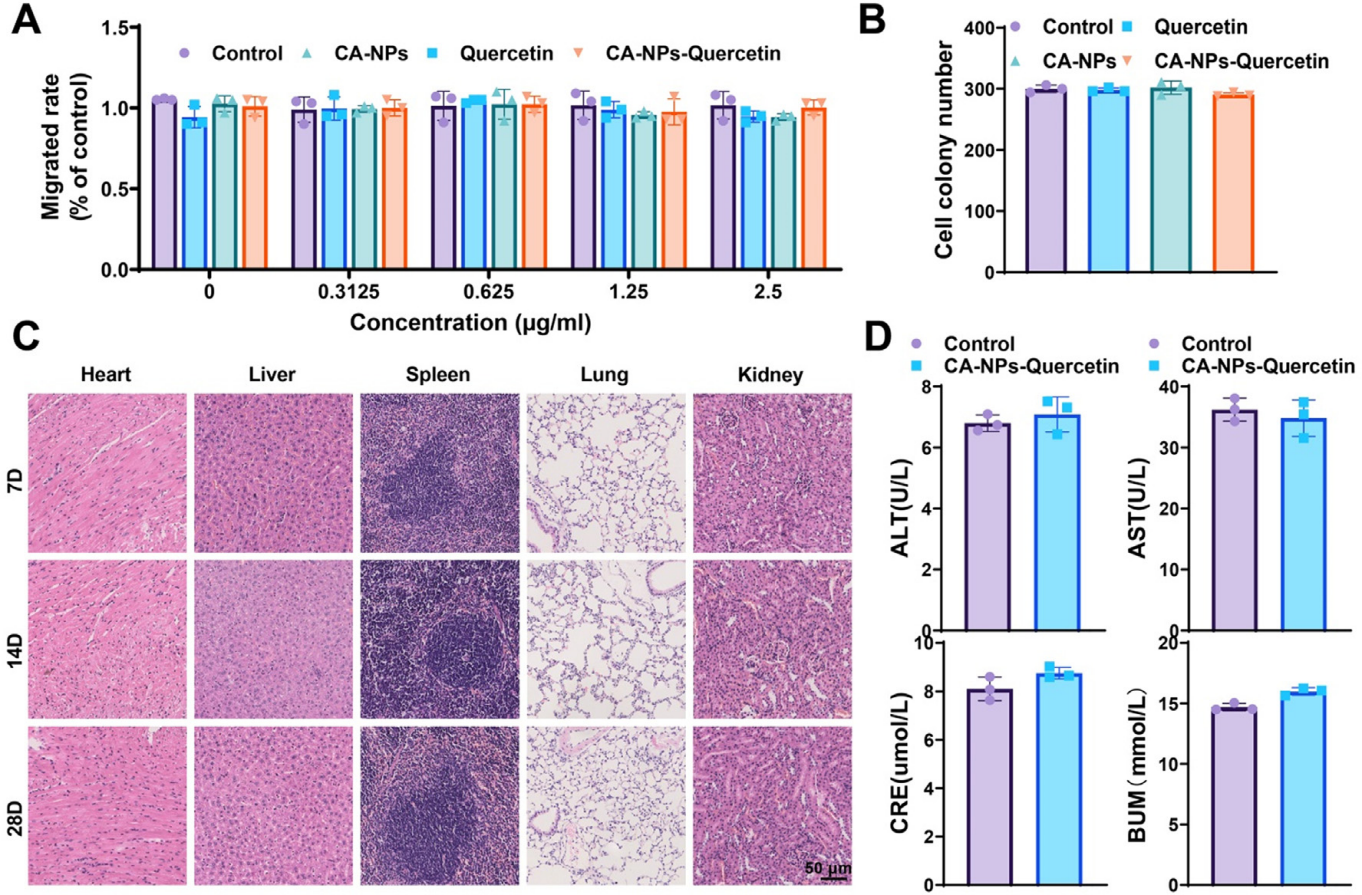
*In vitro* and *in vivo* biocompatibility of CA-NPs-Quercetin. (a) MTT assay evaluating the viability of cells in different groups (concentrations of Quercetin and CA-NPs-Quercetin in the x axis: 0, 0.3125, 0.625, 1.25, and 2.5 μg/ml are the concentrations of Quercetin in both Quercetin and CA-NPs-Quercetin, as well as the concentrations of CA-NPs). (b) Colony formation assay assessing the proliferation capability of cells in each group. (c) Histopathological examination of heart, liver, kidney, spleen, and lung tissues in mice from each group. (d) Biochemical analysis of serum ALT, AST, BUN, and Cr levels in mice from different groups.

*In vitro*, the cytotoxicity of CA-NPs-Quercetin on normal HASMCs was assessed using the 3-(4,5-dimethylthiazol-2-yl)-2,5-diphenyltetrazolium bromide (MTT) proliferation assay and colony formation assay. Cells were divided into four groups: control group, Quercetin group, CA-NPs group, and CA-NPs-Quercetin group. The cells were treated with 0, 0.3125, 0.625, 1.25, and 2.5 μg/ml of the respective compounds, and cell viability was measured at 24 h. The results showed that the CA-NPs-Quercetin group maintained a cell survival rate of over 85% at all concentrations, comparable to the control group, and formed distinct cell colonies, indicating that CA-NPs-Quercetin exhibits low toxicity toward normal cells [Figs. 2(a) and 2(b)].

In the *in vivo* experiment, healthy adult mice were administered CA-NPs-Quercetin via tail vein injection. Blood samples and major organs (heart, liver, spleen, lungs, and kidneys) were collected on days 7, 14, and 28 for histopathological examination and biochemical analysis. Histopathological examination revealed no significant pathological changes, and the organ structures remained normal after CA-NPs-Quercetin treatment [Fig. 2(c)]. These findings suggest that CA-NPs-Quercetin exhibits low cytotoxicity and good biocompatibility both *in vitro* and *in vivo*.

Serum biochemical markers, including liver and kidney function, are crucial for assessing the biotoxicity of nanoparticles. To evaluate the impact of CA-NPs-Quercetin on liver function, alanine aminotransferase (ALT) and aspartate aminotransferase (AST) levels were measured in mice 21 days after injection. Kidney function was assessed by analyzing blood urea nitrogen (BUN) and creatinine (CRE) levels. The results showed that serum ALT, AST, BUN, and CRE levels in the CA-NPs-Quercetin group were within the normal range, with no significant differences compared to the control group, indicating that CA-NPs-Quercetin does not induce toxicity in healthy mice [Fig. 2(d)].

In conclusion, CA-NPs-Quercetin demonstrated excellent biocompatibility both *in vitro* and *in vivo*, with low toxicity to normal cells and mice. Additionally, it did not trigger significant inflammatory responses, underscoring its safety and potential for biomedical applications.

### CA-NPs-Quercetin inhibits the proliferation and migration of HASMCs under asthmatic conditions

**FIG. 3.**
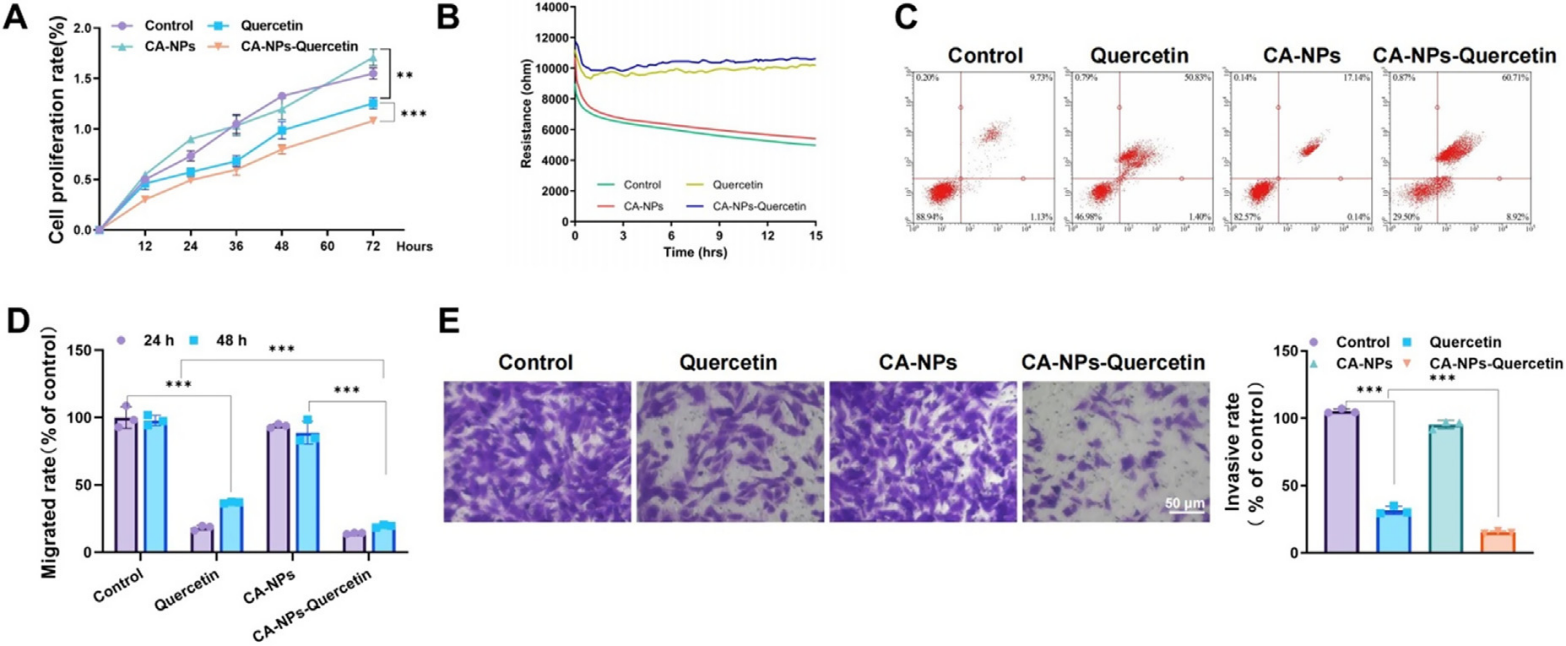
*In vitro* mechanistic studies of CA-NPs-Quercetin on HASMCs. (a) CCK-8 assay measuring the proliferation rates of cells in each group at 12, 24, 36, 48, 60, and 72 h. (b) ECIS system monitoring real-time cell proliferation at 3, 6, 9, 12, and 15 h. (c) Flow cytometry analysis of apoptosis rates in each group. (d) Wound healing assay assessing the migration ability of cells in each group with images captured at 0, 24, and 48 h. (e) Transwell invasion assay evaluating the invasive capacity of cells in each group. ^*^ denotes significant differences between groups, ^***^p < 0.001.

*In vitro* mechanistic studies were conducted to assess the effect of CA-NPs-Quercetin on the proliferation and migration of HASMCs under simulated asthmatic conditions. HASMCs were stimulated with 30 ng/ml Platelet-Derived Growth Factor-BB (PDGF-BB) for 24 h to induce cell proliferation and migration, mimicking the pathological state of asthma. The cells were divided into four groups: control, Quercetin, CA-NPs, and CA-NPs-Quercetin. Cell proliferation was evaluated using the Cell Counting Kit-8 (CCK-8) assay. The results showed that the proliferation rate of the CA-NPs-Quercetin group was significantly reduced compared to the control group [Fig. 3(a)].

Additionally, real-time monitoring of cell proliferation was performed using an electric cell-substrate impedance sensing (ECIS) system. Healthy, adherent cells generate higher resistance values as they cover more of the electrode surface, while dead or detached cells cause a decrease in resistance. The results demonstrated that the resistance values in the CA-NPs-Quercetin group were significantly higher than those in the other three groups, further confirming the inhibitory effect of CA-NPs-Quercetin on HASMC proliferation [Fig. 3(b)].

Flow cytometry was used to assess apoptosis in each group, and the results showed that the apoptosis rate in the CA-NPs-Quercetin group was significantly higher than that in the control group [Fig. 3(c)]. Additionally, the scratch wound healing assay and Transwell invasion assay were used to evaluate the migration ability of the cells. The scratch assay results indicated that the cell migration rate in the CA-NPs-Quercetin group was significantly lower than that in the control group, with the scratch closure rate reduced after 24 h [Fig. 3(d)]. The Transwell invasion assay showed that the number of invasive cells in the CA-NPs-Quercetin group was reduced compared to the control group [Fig. 3(e)].

In conclusion, CA-NPs-Quercetin significantly inhibited the proliferation and migration of HASMCs *in vitro* and markedly increased the rate of apoptosis. These results provide strong evidence supporting the potential application of CA-NPs-Quercetin in the treatment of airway remodeling in asthma.

### CA-NPs-Quercetin regulates the Hippo-YAP/TAZ pathway

Previous studies have shown that the Hippo-YAP/TAZ signaling pathway plays a critical role in airway remodeling in asthma.^12,25^ Moreover, Quercetin has been reported to inhibit mesangial cell proliferation in early diabetic nephropathy through the Hippo pathway.^26^ To investigate whether CA-NPs-Quercetin affects asthma airway remodeling by modulating the Hippo-YAP/TAZ pathway, we examined the expression of Hippo-YAP/TAZ signaling-related genes (TEAD) and proteins [YAP, TAZ, TEAD, phosphorylated YAP (p-YAP), and phosphorylated TAZ (p-TAZ)] in control, Quercetin, CA-NPs, and CA-NPs-Quercetin groups.

**FIG. 4.**
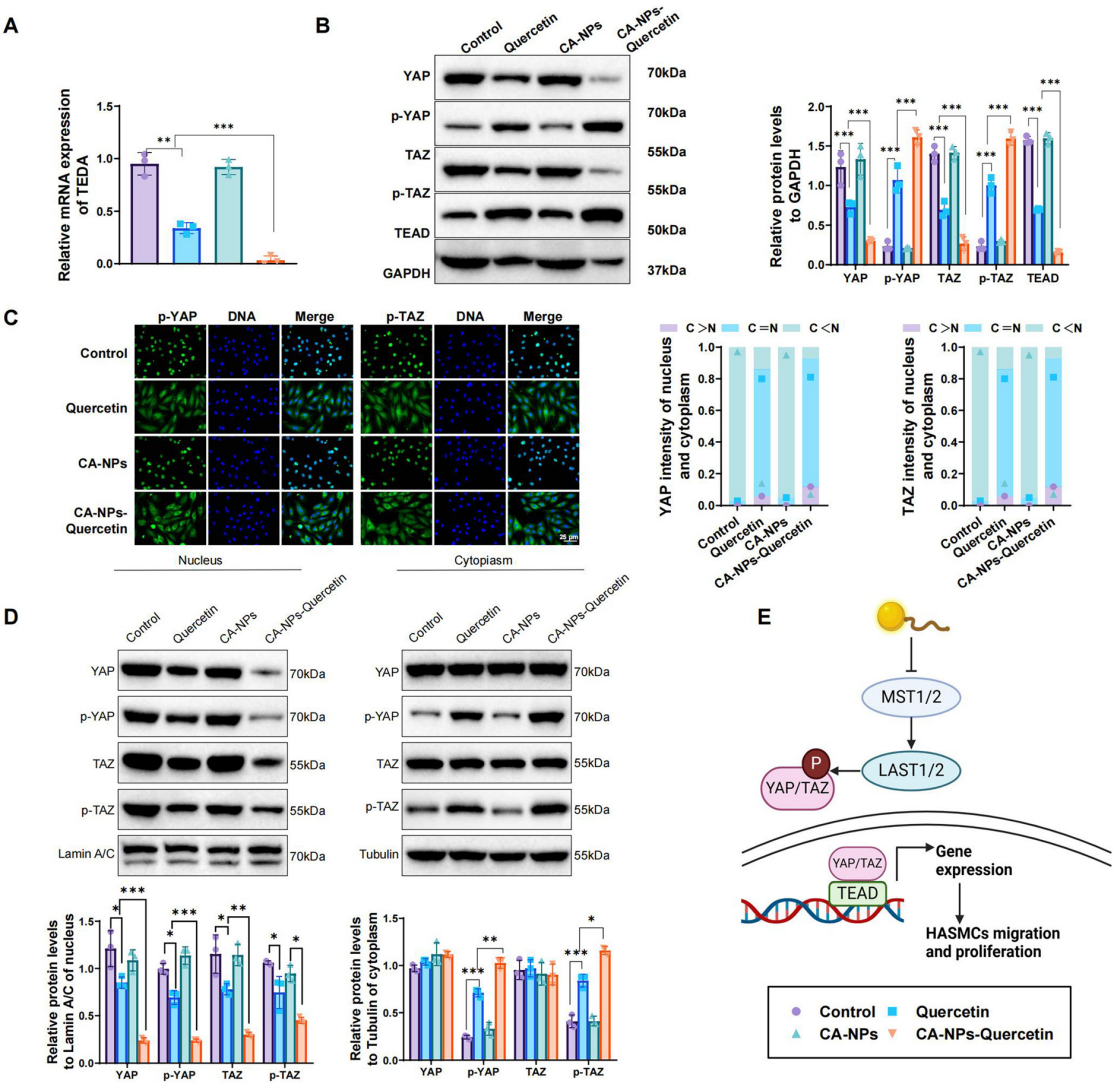
*In vitro* mechanistic studies of CA-NPs-Quercetin on the regulation of the Hippo-YAP/TAZ pathway. (a) RT-qPCR analysis showing the expression levels of TEAD, a key gene regulating cell proliferation and migration in the Hippo-YAP/TAZ signaling pathway, in each group. (b) Western blot analysis of Hippo-YAP/TAZ signaling-related proteins, including YAP, TAZ, TEAD, p-YAP, and p-TAZ, in each group. (c) Immunofluorescence staining showing the cellular localization of YAP and TAZ. (d) Expression levels of YAP, TAZ, p-YAP, and p-TAZ in the cytoplasm and nucleus. (e) Schematic illustration of how CA-NPs-Quercetin activates the Hippo signaling pathway to inhibit the proliferation and migration of HASMCs. ^*^ denotes significant differences between groups, ^**^p < 0.01, ^***^p < 0.001.

RT-qPCR results demonstrated that TEAD mRNA expression was significantly downregulated in the CA-NPs-Quercetin group compared to the other three groups [Fig. 4(a)]. Western blot analysis revealed that the protein expression levels of YAP, TAZ, and TEAD were significantly increased in the CA-NPs-Quercetin group, while the levels of p-YAP and p-TAZ were notably decreased [Fig. 4(b)].

Furthermore, immunofluorescence staining and cellular fractionation were used to assess the localization of YAP and TAZ in the cells. The results showed that, compared to the other groups, YAP and TAZ predominantly accumulated in the cytoplasm in the CA-NPs-Quercetin group, and their phosphorylation levels were significantly increased [Figs. 4(c) and 4(d)]. Figure 4(e) illustrates that CA-NPs-Quercetin activates the Hippo signaling pathway, inhibiting the proliferation and migration of HASMCs by promoting the phosphorylation of YAP and TAZ.

In conclusion, CA-NPs-Quercetin modulates the Hippo signaling pathway by increasing the phosphorylation of YAP and TAZ, leading to their retention in the cytoplasm and significantly reducing TEAD expression. This effectively inhibits the proliferation and migration of HASMCs.

### Inhibition of the Hippo-YAP/TAZ pathway reverses the effects of CA-NPs-Quercetin on HASMC proliferation and migration under asthmatic conditions

**FIG. 5.**
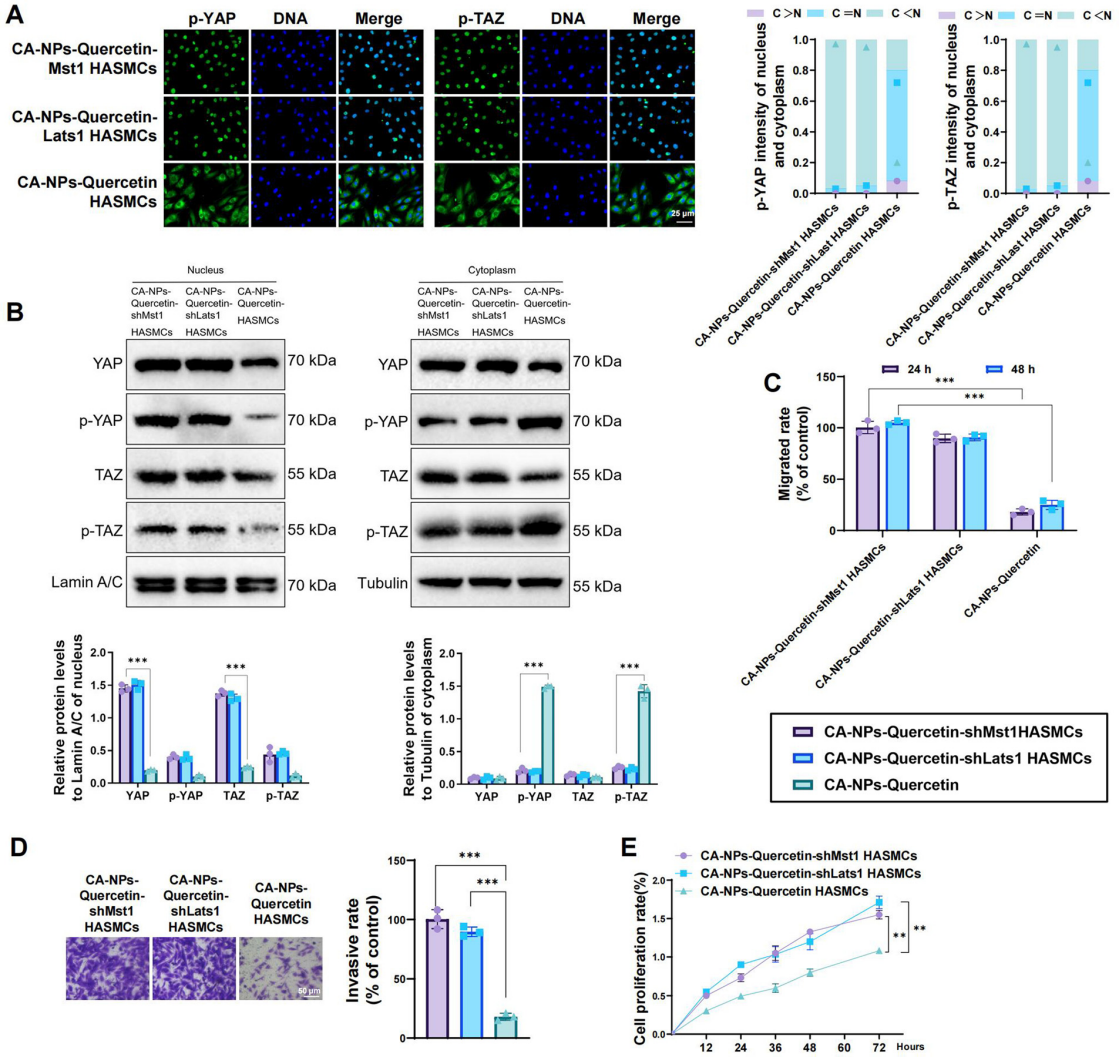
Effects of shMst1 and shLats1 on CA-NPs-Quercetin-induced proliferation and migration of HASMCs. (a) Immunofluorescence staining showing the localization of YAP and TAZ in cells. (b) Expression levels of YAP, TAZ, p-YAP, and p-TAZ in the cytoplasm and nucleus. (c) Wound healing assay assessing the migration ability of cells in each group, with images captured at 0, 24, and 48 h. (d) Transwell invasion assay evaluating the invasive capacity of cells in each group. (e) CCK-8 assay measuring the proliferation rates of cells in each group at 12, 24, 36, 48, 60, and 72 h. ^*^ denotes significant differences between groups, ^**^p < 0.01, ^***^p < 0.001.

Using RNA interference technology, the kinase proteins Mst1 and Lats1 were knocked down in HASMCs. The cells were then stimulated with 30 ng/ml PDGF-BB for 24 h to induce asthmatic conditions, resulting in shMst1 HASMCs and shLats1 HASMCs, which were subsequently co-incubated with CA-NPs-Quercetin for another 24 h (Fig. S3A). RT-qPCR and western blot showed that TEAD expression increased, and the dephosphorylation of YAP and TAZ was enhanced in the Mst1 and Lats1 knockdown cells (Figs. S3B and 3C). Immunofluorescence staining, cellular fractionation, and western blot results further revealed that, compared to the CA-NPs-Quercetin group, both the CA-NPs-Quercetin-shMst1 HASMCs and CA-NPs-Quercetin-shLats1 HASMCs groups exhibited significantly reduced levels of p-YAP and p-TAZ in the cytoplasm. Additionally, YAP and TAZ were notably translocated and accumulated in the nucleus [Figs. 5(a) and 5(b)].

Further assessment of cell migration ability was conducted using the wound healing assay and Transwell invasion assay. The wound healing assay results showed that the migration rate of cells in the CA-NPs-Quercetin-shMst1 HASMC and CA-NPs-Quercetin-shLats1 HASMC groups was significantly higher than that of the CA-NPs-Quercetin group, with the wound closure rate increasing after 24 h [Fig. 5(c)]. The Transwell invasion assay results indicated that the number of invasive cells in the CA-NPs-Quercetin-shMst1 HASMC and CA-NPs-Quercetin-shLats1 HASMC groups was significantly higher than that in the CA-NPs-Quercetin group, showing an increase [Fig. 5(d)].

The CCK-8 experiments revealed a significant increase in cell proliferation in the CA-NPs-Quercetin-shMst1 HASMC group and the CA-NPs-Quercetin-shLats1 HASMC group. In comparison to the control group, cell proliferation rates gradually decreased over time [Fig. 5(e)].

In summary, inhibiting the key kinases in the Hippo-YAP/TAZ pathway can reverse the effects of CA-NPs-Quercetin, promoting the dephosphorylation of YAP and TAZ, leading to their translocation into the nucleus, and thereby restoring the proliferation and migration capabilities of HASMCs.

### CA-NPs-Quercetin significantly improves airway inflammation and remodeling in asthmatic mice

**FIG. 6.**
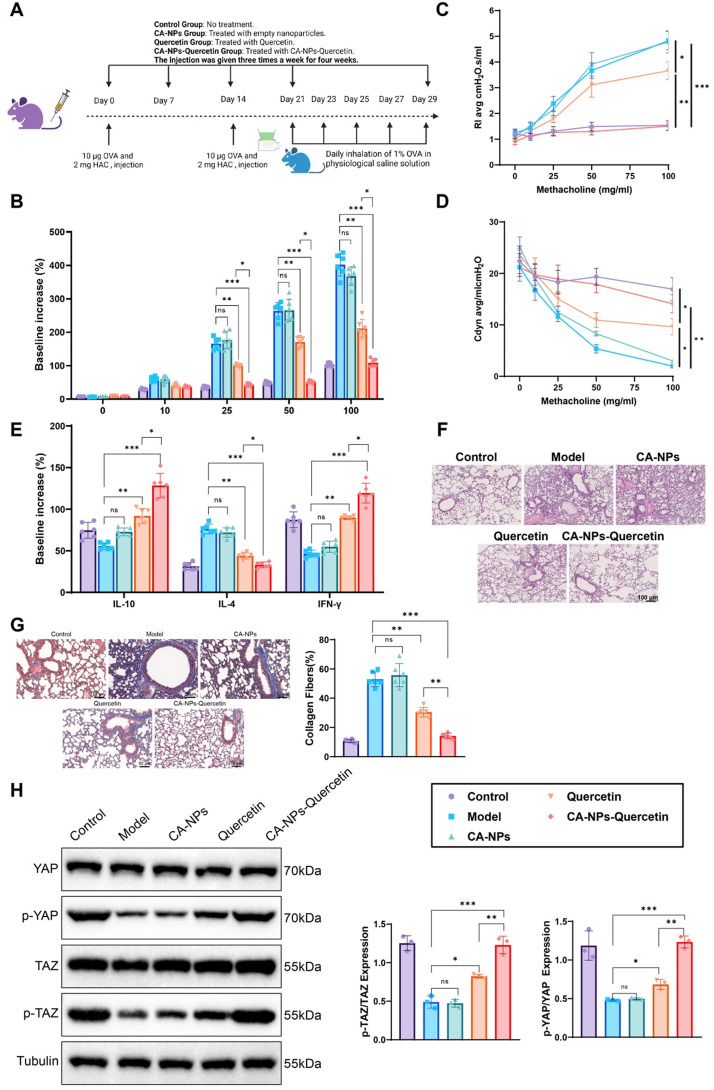
Effects of CA-NPs-Quercetin on airway responsiveness, lung function, and airway remodeling in asthmatic mice. (a) Schematic representation of the animal study protocol. (b) Changes in airway responsiveness in asthmatic mice following acetylcholine challenge. (c) and (d) Changes in resistance and compliance in mice under acetylcholine stimulation. (e) Expression levels of inflammatory cytokines in mice. (f) H&E staining showing the pathological changes in lung tissues of asthmatic mice after treatment. (g) MASSON staining showing collagen deposition in lung tissue, where collagen fibers appear blue, muscle fibers and cytoplasm appear red, and nuclei appear blue-black. Scale bar = 50 μm. (h) Western blot analysis of YAP and TAZ expression levels in lung tissues; ^*^ denotes significant differences between groups, ns indicates *p* > 0.05, ^*^p < 0.05, ^**^p < 0.01, ^***^p < 0.001.

The experimental design is illustrated in Fig. 6(a). In the asthma model group, airway responsiveness was significantly increased, indicating heightened sensitivity to acetylcholine stimulation [Fig. 6(b)]. Compared to the control group, lung function in the model, CA-NPs, and Quercetin groups was markedly impaired, with a significant increase in airway resistance (RI) and a decrease in lung compliance (Crs). In contrast, the CA-NPs-Quercetin group showed a notable reduction in airway responsiveness, and lung function was significantly improved, with RI and Crs values approaching those of the control group [Figs. 6(c) and 6(d)]. These findings suggest that CA-NPs-Quercetin exerts a protective effect on airway responsiveness and lung function in asthmatic mice.

The bronchoalveolar lavage fluid (BALF) analysis results showed that the total number of inflammatory cells in the model group mice was significantly increased, especially eosinophils. In contrast, the total number of inflammatory cells and eosinophils in the BALF of the CA-NPs-Quercetin group was significantly reduced. Additionally, the inflammatory cytokine IL-4 level was significantly elevated in the model group, while IL-10 and IFN-γ levels were significantly decreased [Fig. 6(e)]. In the CA-NPs-Quercetin group, IL-4 expression was reduced compared to the control group (56.65%), while IL-10 (130.91%) and IFN-γ (156.58%) levels were significantly upregulated [Fig. 6(e)]. These results suggest that CA-NPs-Quercetin effectively inhibits airway inflammation.

Histopathological examination revealed significant airway remodeling in the lung tissue of the model group mice, including epithelial hyperplasia and smooth muscle thickening [Fig. 6(f)]. In comparison, the lung tissue pathology in the CA-NPs-Quercetin group showed marked improvement, with a significant reduction in epithelial hyperplasia and smooth muscle thickening [Fig. 6(f)].

Masson's staining was performed to assess collagen deposition in the lung tissue of the mice for a more comprehensive histopathological analysis. The results showed that compared to the Model group, collagen deposition in the lung tissue of the Quercetin (42.53%) and CA-NPs-Quercetin (73.20%) groups was significantly reduced. Additionally, compared to the Quercetin group, the CA-NPs-Quercetin group also exhibited a significant reduction in lung tissue collagen deposition [Fig. 6(g)]. These results indicate that CA-NPs-Quercetin has a significant effect in alleviating asthma-related airway remodeling.

Western blot results showed a downregulation of phosphorylated YAP and TAZ levels in the lung tissues of the asthma model group [Fig. 6(h)]. In contrast, the CA-NPs-Quercetin group exhibited a significant upregulation of YAP and TAZ phosphorylation, indicating activation of the Hippo signaling pathway.

The *in vivo* study further demonstrated that CA-NPs-Quercetin effectively inhibited airway remodeling in asthma by suppressing the proliferation and promoting the apoptosis of airway smooth muscle cells.

### Mechanism of CA-NPs-Quercetin in activating the Hippo pathway to prevent asthmatic airway remodeling *in vivo*

**FIG. 7.**
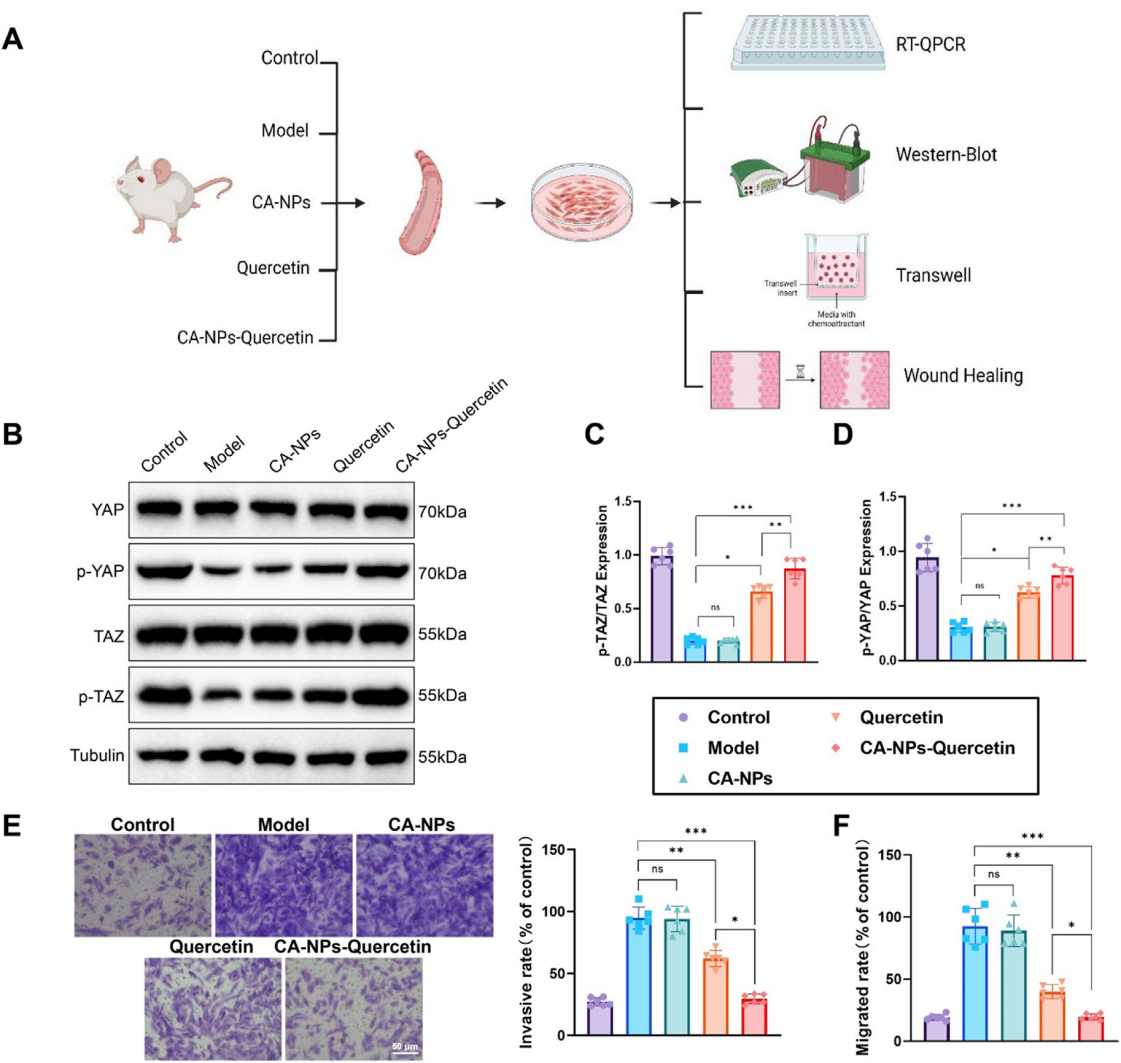
Activation of the Hippo signaling pathway by CA-NPs-Quercetin *in vivo*. (a) Schematic representation of the primary cell isolation and experimental protocol in mice. (b)–(d) Western blot analysis showing the phosphorylation levels of YAP and TAZ in primary airway smooth muscle cells from mice. (e) Transwell migration assay evaluating the migration capacity of primary airway smooth muscle cells in each group of mice. (f) Wound healing assay assessing the migration ability of primary airway smooth muscle cells in mice. ^*^ denotes significant differences between groups, ns indicates *p* > 0.05, ^*^p < 0.05, ^**^p < 0.01, ^***^p < 0.001.

To further investigate the mechanism by which CA-NPs-Quercetin prevents airway remodeling in asthma by activating the Hippo signaling pathway, primary airway smooth muscle cells were isolated and cultured from mice in the control, model, CA-NPs, Quercetin, and CA-NPs-Quercetin groups. The experimental workflow is outlined in Fig. 7(a).

Western blot was used to detect the phosphorylation levels of Hippo pathway-related proteins (YAP and TAZ) in the primary airway smooth muscle cells from each group. The results showed that the phosphorylation levels of YAP and TAZ were significantly upregulated in the CA-NPs-Quercetin group. Compared to the control group, both the phosphorylation levels of YAP and TAZ significantly increased in the CA-NPs-Quercetin group [Figs. 7(b)–7(d)].

The migration ability of primary airway smooth muscle cells from each group of mice was assessed using the Transwell migration assay. The results indicated that the number of migrating cells in the CA-NPs-Quercetin group was significantly lower than that in the control group [Fig. 7(e)]. Additionally, the wound healing assay further confirmed this finding, with the CA-NPs-Quercetin group showing a significantly reduced cell migration rate, and the wound closure rate decreased after 24 h [Fig. 7(f)].

In conclusion, both *in vitro* and *in vivo* experiments demonstrated that CA-NPs-Quercetin significantly activated the Hippo signaling pathway, inhibiting the proliferation and migration of airway smooth muscle cells while promoting apoptosis. These results suggest that CA-NPs-Quercetin effectively prevents asthmatic airway remodeling by regulating the Hippo signaling pathway, providing strong data to support its potential application in asthma treatment.

## DISCUSSION

This study demonstrates that CA-NPs-Quercetin, by activating the Hippo signaling pathway, effectively inhibits the proliferation and migration of airway smooth muscle cells. This finding is consistent with previous research on the anti-proliferative effects of Quercetin, but it introduces a significant mechanistic innovation. Traditional studies have primarily focused on Quercetin's broad anti-inflammatory and antioxidant properties as a natural flavonoid. In contrast, this study is the first to combine nanoparticle technology, significantly enhancing the bioavailability and stability of Quercetin.^27^ Compared to studies applying Quercetin directly, our research shows that CA-NPs-Quercetin achieved an EE of 84 ± 2%, effectively overcoming the issue of Quercetin's poor water solubility. This enhancement led to a more pronounced effect on airway smooth muscle cells, particularly in inhibiting proliferation, where it exhibited superior efficacy.

In terms of inhibiting airway smooth muscle cell migration, this study presents findings that differ significantly from previous research. Earlier studies primarily focused on using anti-inflammatory drugs or inhibiting signaling pathways to block cell migration. In contrast, this study inhibited airway smooth muscle cell migration by activating the Hippo signaling pathway, increasing the phosphorylation of YAP and TAZ, and promoting their retention in the cytoplasm. The migration rate was significantly reduced after 24 h, suggesting that the Hippo pathway may be a key regulatory mechanism in preventing asthmatic airway remodeling. Notably, other existing asthma treatments have not demonstrated such a clear effect on the Hippo pathway, highlighting the potential of CA-NPs-Quercetin as an innovative therapeutic strategy targeting this pathway.

The uniqueness of the Hippo signaling pathway is thoroughly demonstrated in this study. Unlike traditional pathways such as TGF-β and Wnt, the Hippo pathway regulates cell proliferation and migration primarily through the phosphorylation of YAP/TAZ rather than by inhibiting inflammatory responses.^28^ This distinct mechanism makes the Hippo pathway particularly significant in the context of airway smooth muscle cells. In this study, the activation of the Hippo pathway not only effectively inhibited cell proliferation and migration but also reduced the expression of pro-proliferative genes by restricting the nuclear translocation of YAP/TAZ. This highlights the potential of the Hippo pathway as a novel therapeutic target for intervening in asthmatic airway remodeling.

The innovative application of CA-NPs is another key highlight of this study. Nanotechnology has garnered significant attention in drug delivery systems in recent years, and this study successfully employed CA-NPs to load Quercetin, utilizing physical adsorption to achieve efficient drug delivery. Compared to traditional drug delivery systems, CA-NPs offer superior biocompatibility and targeting ability while also significantly enhancing Quercetin's efficacy by improving its stability. This technology holds great potential for application in respiratory diseases, not only in asthma treatment but also in other conditions related to airway remodeling, such as chronic obstructive pulmonary disease (COPD). Quercetin, as a natural flavonoid, has poor hydrophobicity and metabolic stability. Chitosan-alginate nanoparticles, through their nanoscale encapsulation effect and good solubility, significantly enhance Quercetin's stability, delay its degradation, and extend its action time in the cellular microenvironment. Chitosan-alginate nanoparticles exhibit good biocompatibility and adhesion, allowing them to interact electrostatically with the negatively charged cell membranes of HASMCs, promoting nanoparticle uptake. After loading Quercetin, the drug can achieve sustained release through swelling/degradation mechanisms, maintaining effective concentrations of Quercetin in the cellular microenvironment. Compared to free Quercetin, nanoparticles prevent rapid drug metabolism, prolonging its action time. Chitosan and alginate themselves also have certain antioxidant and anti-inflammatory properties, which may synergize with Quercetin to enhance the overall effect.^29,30^ In summary, Quercetin-loaded chitosan-alginate nanoparticles significantly enhance its inhibitory effects on PDGF-BB-stimulated HASMCs through improved stability, bioavailability, targeted delivery, sustained release, and synergistic anti-inflammatory effects.

The analysis of both *in vitro* and *in vivo* experimental results reveals a strong consistency between the two. *In vitro*, CA-NPs-Quercetin significantly inhibited the proliferation and migration of airway smooth muscle cells. *In vivo* experiments on the mouse asthma model demonstrated that CA-NPs-Quercetin effectively improved airway responsiveness reduced the total number of inflammatory cells in BALF, and decreased airway smooth muscle proliferation and thickness. These *in vivo* findings further validated the efficacy observed *in vitro*, indicating that this nanoparticle complex plays a significant role in treating airway remodeling in asthma. Additionally, the study's analysis of Hippo signaling pathway-related protein expression in lung tissues further supports the critical regulatory function of this pathway *in vivo*.

Compared to existing asthma treatments, CA-NPs-Quercetin demonstrates several advantages. Traditional anti-asthma drugs, such as glucocorticoids and leukotriene inhibitors, are effective at alleviating symptoms and controlling inflammation but have a limited impact on airway remodeling. In contrast, the results of this study show that CA-NPs-Quercetin not only inhibits the abnormal proliferation and migration of airway smooth muscle but also prevents airway remodeling by activating the Hippo pathway. This offers a novel approach for developing more targeted asthma therapies in the future, particularly in combination with existing treatment options, where CA-NPs-Quercetin holds significant potential for synergistic applications.

Finally, this study conducted a preliminary evaluation of the safety and tolerability of CA-NPs-Quercetin. In the *in vivo* experiments via tail vein injection, no significant toxic reactions were observed, indicating that the nanoparticle complex has good biocompatibility. Compared to other nanomedicines used in respiratory diseases, CA-NPs-Quercetin demonstrates a certain advantage in terms of safety. However, although the results from animal experiments are promising, further preclinical studies and toxicological assessments are necessary to evaluate its long-term safety and feasibility, ensuring that it can be successfully applied in clinical treatments in the future.

This study successfully inhibited the proliferation and migration of airway smooth muscle cells and prevented asthmatic airway remodeling by constructing CA-NPs-Quercetin and activating the Hippo signaling pathway. For the first time, this research combined nanotechnology with a natural anti-inflammatory agent, significantly enhancing Quercetin's bioavailability and stability, overcoming the limitations of its traditional application. Moreover, the study revealed the critical role of the Hippo signaling pathway in regulating the pathological processes of airway smooth muscle cells, offering new insights for developing targeted therapies for airway remodeling. The nanoparticle complex notably improved pathological features in a mouse model of asthma, indicating its potential clinical application in asthma treatment. Beyond inhibiting airway remodeling, CA-NPs-Quercetin may reduce airway inflammation, improve lung function, and enhance long-term outcomes for patients.

Despite the promising potential of CA-NPs-Quercetin in asthma treatment demonstrated in this study, several limitations remain. First, the research was conducted solely in a mouse model, and further evaluation is needed in larger-scale animal models and preclinical trials to assess its safety and efficacy comprehensively. Second, although Quercetin possesses multiple bioactivities, its metabolic pathways in complex physiological environments are not yet well understood, which may impact its clinical effectiveness. Additionally, this study primarily focused on the regulation of the Hippo signaling pathway, and future research should investigate the nanoparticle's effects on other potential signaling pathways. Future studies should focus on expanding the application of CA-NPs-Quercetin, particularly exploring its potential in other chronic inflammatory diseases. Moreover, optimizing the nanoparticle preparation process to enhance its *in vivo* targeting capability and drug delivery efficiency may be crucial. An in-depth analysis of Quercetin's mechanism of action using multi-omics technologies can provide a more comprehensive understanding of its biological effects, facilitating the development of therapeutic strategies with greater clinical translation potential. With the advancement of clinical trials, CA-NPs-Quercetin holds the promise of becoming an effective therapeutic option for asthma and other chronic respiratory diseases.

## CONCLUSION

In this study, CA-NPs-Quercetin was successfully synthesized and characterized, and it was demonstrated to effectively regulate the proliferation, migration, and apoptosis of HASMCs both *in vitro* and *in vivo*. The *in vitro* experiments showed that CA-NPs-Quercetin activated the Hippo signaling pathway, increasing the phosphorylation levels of YAP and TAZ, thereby inhibiting HASMC proliferation and migration and ultimately preventing asthmatic airway remodeling. *In vivo* experiments further validated the therapeutic potential of this nanoparticle in a mouse asthma model, significantly improving airway reactivity, lung function, and airway remodeling. These experimental results collectively show that CA-NPs-Quercetin has good biocompatibility and low toxicity. The efficient targeted delivery of nanoparticles reduces systemic side effects and enhances local therapeutic effects, laying the foundation for precision medicine. Future research will focus on toxicological evaluations, large animal validations, and delivery optimization to accelerate its transition from laboratory to clinical use. CA-NPs-Quercetin demonstrates great potential in asthma treatment and could become an innovative drug for treating asthma and related diseases.

## METHODS

### Preparation of CA-NPs

To prepare CA-NPs, we utilized the ionic cross-linking method. First, chitosan oligosaccharide (Sigma-Aldrich, USA) was dissolved in 1% acetic acid solution (Fisher Scientific, USA) and stirred until fully dissolved. Then, alginic acid (Sigma-Aldrich, USA) was dissolved in de-ionized water (Millipore, USA). Subsequently, the chitosan oligosaccharide solution and alginic acid solution were mixed in a 1:1 volume ratio, and sodium tripolyphosphate (TPP, Sigma-Aldrich, USA) was slowly added to form stable nanoparticles. The reaction mixture was stirred at room temperature for 2 h to ensure complete cross-linking.

### Loading of Quercetin

To load Quercetin, the physical adsorption method was employed. Quercetin (Sigma-Aldrich, USA) was dissolved in ethanol (Fisher Scientific, USA) and then added to the CA-NPs solution. The mixture was stirred for 24 h. Following this, the solution was centrifuged at 3000 rpm for 10 min (Centrifuge 5810R, Eppendorf, Germany) to collect the Quercetin-loaded CA-NPs (CA-NPs-Quercetin). The resulting particles were washed three times with de-ionized water to remove any unadsorbed Quercetin.

### Transmission electron microscopy (TEM)

After preparing the nanoparticle suspension, CA-NPs and CA-NPs-Quercetin were dispersed in de-ionized water. A small amount of the nanoparticle suspension was placed onto a copper grid coated with a carbon film and allowed to air-dry at room temperature, ensuring the nanoparticles adhered firmly to the grid. The prepared copper grid was then mounted onto the TEM sample holder. The transmission electron microscope (JEM-2100, JEOL, Japan) was operated at an accelerating voltage of 200 kV to ensure high-resolution imaging. The morphology of the nanoparticles was observed, and multiple images were captured to provide a comprehensive view of their structure. TEM images were analyzed to determine the size, shape, and distribution of both CA-NPs and CA-NPs-Quercetin, with particular attention to the morphological differences between unloaded and Quercetin-loaded nanoparticles to confirm successful loading and assess the potential impact of the loading process on nanoparticle structure.

### Zeta potential measurement by laser particle size analyzer

The Zeta potential and PDI (polydispersity index) of the nanoparticles were measured using a laser particle size analyzer (Zetasizer Nano ZS, Malvern Instruments, UK). First, CA-NPs and CA-NPs-Quercetin were each suspended in de-ionized water to prepare sample solutions with a concentration of 1 mg/ml. The samples were then subjected to ultrasonic treatment for 5 min using an ultrasonic processor (Branson Ultrasonics, USA) to ensure adequate dispersion of the particles. The laser particle size analyzer was set to a measurement temperature of 25 °C, and once the instrument was stabilized, the measurement process began. The samples were carefully injected into the measurement cell (DTS1070, Malvern Instruments, UK), ensuring no air bubbles were present, and the cell was placed in the instrument's sample holder. Each sample was measured three times, with a 1-min interval between each measurement to ensure reproducibility. The instrument automatically calculated and displayed the Zeta potential and PDI (polydispersity index) for each measurement, and the average value of the three measurements was recorded as the final result. These steps ensured the Zeta potential and PDI measurement was accurate and reliable for each sample.^31^

### UV–visible spectrophotometry (UV–Vis) and high-performance liquid chromatography (HPLC) analysis

The encapsulation efficiency (EE) and loading capacity (LC) of Quercetin were assessed using a UV–visible spectrophotometer (UV–Vis, Cary 60, Agilent Technologies, USA). The absorbance of the samples was measured at 370 nm, and the concentration of Quercetin was calculated based on a standard curve. For a more precise evaluation of Quercetin loading efficiency, HPLC (1260 Infinity II, Agilent Technologies, USA) was employed. The HPLC system was equipped with a C18 column (4.6 mm × 250 mm, 5 μm, Agilent Technologies, USA), with a mobile phase consisting of methanol and water (70:30, v/v). The flow rate was set at 1.0 ml/min, and detection was carried out at a wavelength of 370 nm. These two analytical techniques were used in combination to ensure accurate and reliable measurement of Quercetin's EE and LC.

### Fourier transform infrared spectroscopy (FTIR) and thermogravimetric analysis (TGA)

FTIR (Nicolet iS50, Thermo Fisher Scientific, USA) was used to confirm the loading of Quercetin onto the nanoparticles. The samples were mixed with KBr and pressed into pellets, and the infrared spectrum was recorded within the scanning range of 4000–400 cm^−1^, the number of scans set to 32, and the resolution set to 4 cm^−1^ to obtain the infrared spectrum to obtain clear spectral data. Subsequently, TGA (TGA 5500, TA Instruments, USA) was conducted to assess the thermal stability of the nanoparticles. The samples were heated in a nitrogen atmosphere from 25 to 600 °C at a heating rate of 10 °C/min. This analysis helped to evaluate the thermal degradation behavior and stability of the nanoparticles.

### Drug release study

To evaluate the release behavior of Quercetin from CA-NPs, an *in vitro* drug release experiment was conducted under different pH conditions. The CA-NPs-Quercetin suspension was placed in dialysis bags (MWCO 12–14 kDa, Sigma-Aldrich, USA) and immersed in pH 7.4 phosphate buffer (Sigma-Aldrich, USA) and pH 5.5 acetate buffer (Sigma-Aldrich, USA) at 37 °C. Each setup was placed on a thermostatic shaker (Thermo Fisher Scientific, USA) set to 100 rpm. Samples were taken at regular intervals of 0, 1, 2, 4, 6, 12, and 24 h. The concentration of released Quercetin was measured using a UV–visible spectrophotometer (UV–Vis, Cary 60, Agilent Technologies, USA) at a wavelength of 370 nm to determine the drug release kinetics.

### Cell labeling and uptake experiment

CA-NPs-Quercetin was labeled with the green fluorescent lipophilic dye PKH67 (Umibio, UR52303), following the manufacturer's protocol to ensure efficient labeling. After labeling, the nanoparticles were centrifuged at 10 000 × g for 10 min to remove any unbound PKH67 and resuspended in phosphate-buffered saline (PBS). The labeled CA-NPs-Quercetin was then added to HASMCs at 70% confluence (5 × 10^5^ cells per well) in sterile 24-well plates and incubated for 3, 6, and 12 h. At each time point, the cells were washed three times with PBS to remove non-internalized nanoparticles. The treated cells were subsequently fixed on slides using 4% paraformaldehyde (Sigma-Aldrich, USA) for 10 min and washed again three times with PBS. The intracellular distribution of PKH67-labeled CA-NPs-Quercetin was observed under a confocal microscope, and the fluorescence signal was recorded. Finally, cells from each time point were collected, digested with 0.25% trypsin (Gibco, USA), and centrifuged at 300 × g for 5 min. After resuspension in PBS, the nanoparticle uptake was quantified using flow cytometry (BD Biosciences, USA), analyzing the fluorescence signals from at least 10 000 cells per sample.

### CCK-8 assay

Cells from each group were seeded into 96-well plates at a density of approximately 5 × 10^3^ cells per well, with five replicates for each group. After 24 h of incubation, treatments were initiated by adding appropriate concentrations of blank nanoparticles, Quercetin, and CA-NPs-Quercetin to the respective groups. The control group received an equal volume of culture medium. The cells were further incubated for 24, 48, and 72 h. Two hours before the end of each time point, 10 μl of CCK-8 solution (Dojindo Laboratories, Japan) was added to each well, followed by incubation for another 2 h. After incubation, the absorbance of each well was measured at 450 nm using a microplate reader (Bio-Rad, USA). Cell proliferation rates were calculated based on absorbance values, with the control group set as 100% proliferation. The proliferation rates of the different treatment groups at 24, 48, and 72 h were compared to evaluate the effect of CA-NPs-Quercetin on the proliferation of HASMCs.

### Cell incubation with nanoparticles

Cells were seeded into 24-well plates at a density of 6 × 10^5^ cells per well and cultured in 1 ml of Dulbecco’s Modified Eagle Medium (DMEM) medium containing 10% Fetal Bovine Serum (FBS) and 1% PBS. After 24 h of incubation, the culture medium was discarded. Cells were then treated with different concentrations (0, 0.3125, 0.625, 1.25, and 2.5 μg/ml) of Quercetin, CA-NPs, and CA-NPs-Quercetin, and incubated for an additional 24 h. A blank control group was also established for comparative analysis in subsequent experiments.

### RT-qPCR

First, total RNA was extracted from the cells using an RNA extraction kit (Yeasen, China). The extracted RNA was then reverse-transcribed into cDNA using a cDNA synthesis kit (Yeasen, China). Real-time quantitative PCR was performed using RT-PCR SYBR Green Master Mix (Yeasen, China), and the expression levels of target genes were analyzed and compared across groups using the 2^−ΔΔCt^ method. The calculation formula is 2^−ΔΔCt^ = 2^−(ΔCt value^ ^−^ ^average ΔCt of the control group)^, where ΔCt value = target gene Ct value − GAPDH Ct value.

The primer sequences are as follows: TEAD1 (human): Forward 5′-ATTGAGCCCAGCAGCTGGAG-3′, Reverse 5′-TCAGTCCTTTACAAGCCTGTA-3′; YAP (mouse): Forward 5′-CCCTCGTTTTGCCATGAACC-3′, Reverse 5′-ATTCCGTATTGCCTGCCGAA-3′; TAZ (mouse): Forward 5′-ATGGAAAGGATGAGTGACTCTGC-3′, Reverse 5′-TCCCACATGGTGGATAGATAGC-3′; GAPDH (human): Forward 5′-TGCAACCGGGAAGGAAATGA-3′, Reverse 5′-CATCACCCGGAGGAGAAAT-3′; GAPDH (mouse): Forward 5′-CATCACTGCCACCCAGAAGACTG-3′, Reverse 5′-ATGCCAGTGAGCTTCCCGTTCAG-3′.

### Lentivirus and cell transfection

Potential short hairpin RNA (shRNA) target sequences were analyzed based on cDNA sequences from the GenBank database. Two shRNA sequences targeting the Mst and Lats genes were designed, along with a negative control shRNA (sh-NC) that did not contain knockdown sequences. All oligonucleotides were synthesized by GenePharma® (Shanghai, China). Subsequently, the lentiviral packaging system was constructed using the pLKO.1 lentiviral gene silencing vector. Packaging plasmids and the target vector were co-transfected into HEK293T cells (at 80%–90% confluence) using Lipofectamine 3000 (Thermo Fisher Scientific, USA). After 48 h of incubation, the supernatant containing lentiviral particles was collected and filtered through a 0.22 μm filter to remove impurities. Following centrifugation, the viral particles were concentrated in the supernatant. The viral supernatant was then mixed with Lenti-X™ Concentrator (Takara, Japan) at a 3:1 ratio, inverted to mix thoroughly, and incubated overnight at 4 °C. The next day, the mixture was centrifuged at 5000 × g for 30 min, the supernatant was discarded, and the viral pellet was gently resuspended in 100 μl of DMEM by pipetting.

Once HASMCs reached the logarithmic growth phase, they were digested with trypsin and gently resuspended to prepare a cell suspension at a concentration of 1 × 10^6^ cells/ml. The suspension was seeded into 12-well plates. A total of 400 μl serum-free DMEM was added, followed by the addition of 100 μl viral solution and 10 μg/ml polybrene. After 6 h, 500 μl of complete DMEM medium containing 10% FBS and 1% penicillin–streptomycin was added, and the cells were incubated overnight. The following day, cells were collected, centrifuged, and the supernatant discarded. Fresh 1 ml of complete DMEM medium was then added. Stable cell lines were selected by adding 2 μg/ml puromycin (UC0E03, Sigma-Aldrich, Germany) for 2 weeks.

The cell transfection groups were divided as follows: (1) sh-NC group: transfected with the negative control knockdown lentiviral vector; (2) sh-Mst group: transfected with the sh-Mst-1 lentiviral vector; (3) sh-Lats group: transfected with the sh-Lats lentiviral vector.

After 48 h of transfection, RT-qPCR was performed to measure mRNA levels and verify the efficiency of gene knockdown and overexpression. All plasmids used were designed and synthesized by Ribobio (Guangzhou, China).

The primer sequences were as follows: Negative control: 5′-UUC UCC GAA CGU GUC ACG U-3′; sh-Mst: 5′-AGTTGAGTGATAGCTGGGAAA-3′; sh-Lats1: 5′-GAAGATAAAGACACTAGGAAT-3′.

### Western blot

When the cell confluence reached approximately 80%, the culture medium was discarded, and the cells were washed twice with PBS. Radioimmunoprecipitation Assay (RIPA) lysis buffer (Beyotime, China) mixed with protease inhibitors (Roche, Switzerland) was added, and the cells were lysed on ice for 30 min. The lysate was then centrifuged at 12 000 × g for 15 min at 4 °C, and the supernatant was collected. Protein concentration was determined using a BCA protein assay kit (Thermo Fisher Scientific, USA) following the manufacturer's instructions. Standard solutions and samples were added to a 96-well plate, followed by the working reagent. After incubation at room temperature for 30 min, absorbance was measured at 562 nm using a microplate reader (Bio-Rad, USA) to calculate protein concentration. Equal amounts of protein (30 μg per well) were mixed with 5× loading buffer and denatured at 100 °C for 5 min. The denatured proteins were then separated by SDS–PAGE. After electrophoresis, proteins were transferred onto polyvinylidene fluoride (PVDF) membranes (Millipore, USA) using 1× transfer buffer containing 20% methanol. The membranes were blocked with 5% nonfat milk (Bio-Rad, USA) at room temperature for 1 h. Primary antibodies (YAP, TAZ, and TEAD; Cell Signaling Technology, USA; 1:1000 dilution) were added, and the membranes were incubated overnight at 4 °C. The next day, the membranes were washed three times with Tris-Buffered Saline with Tween 20 (TBST) for 10 min each. horseradish peroxidase (HRP)-conjugated secondary antibodies (Santa Cruz Biotechnology, USA; 1:2000 dilution) were added, followed by a 1-h incubation at room temperature. The membranes were then washed three more times with TBST for 10 min each. Protein bands were visualized using an ECL detection reagent (Bio-Rad, USA). The ECL reagent was evenly applied to the membranes, and after a 1-min reaction, excess liquid was removed. The membranes were then placed in an imaging system (Bio-Rad, USA), and protein signals were detected by chemiluminescence.

### Electric cell-substrate impedance sensing (ECIS) impedance measurement

Cells were divided into four groups: control group, Quercetin group, CA-NPs group, and CA-NPs-Quercetin group. Each group of cells was seeded onto the electrode area of the ECIS plate at a density of 1 × 10^4^ cells per well. The ECIS system was initiated, and measurement parameters were set to record impedance changes at 3, 6, 9, 12, 15, and 72 h. Impedance variation curves were used to assess the proliferation rate of each group. The ECIS software was employed to analyze the impedance data and generate cell proliferation curves, allowing a comparison of proliferation differences among the treatment groups. This analysis helped to evaluate the impact of CA-NPs-Quercetin on cell proliferation.

### Flow cytometry

Cells from each group were digested using trypsin (Gibco, USA), collected, and centrifuged at 300 × g for 5 min. The supernatant was discarded, and the cells were resuspended in calcium- and magnesium-free PBS (Gibco, USA). To each cell suspension, 5 μl of Annexin V-FITC and 5 μl of propidium iodide (PI) were added, mixed gently, and incubated in the dark at room temperature for 15 min. After incubation, 400 μl of Annexin V binding buffer (BD Biosciences, USA) was added, and the mixture was gently mixed again. The cells were analyzed using a flow cytometer (BD FACSCanto II, BD Biosciences, USA), with FITC and PI channels set to detect fluorescence signals from at least 10 000 cells. Data were processed using FlowJo software (BD Biosciences, USA). Apoptotic cells were identified by double staining with Annexin V/PI, and the percentage of fluorescence-positive cells was used to calculate the apoptosis rate for each group.

### Immunofluorescence staining

To observe the nuclear localization of YAP and TAZ, cells were first seeded in 6-well plates and fixed with 4% paraformaldehyde (Sigma-Aldrich, USA) for 20 min. Cells were then permeabilized on ice for 5 min using PBS containing 0.5% Triton X-100 (Sigma-Aldrich, USA). Following permeabilization, the cells were blocked with 5% Bovine Serum Albumin (BSA) (Yeasen, China) for 30 min and incubated overnight at 4 °C with primary antibodies—rabbit anti-YAP and rabbit anti-TAZ (Cell Signaling Technology, USA; 1:1000 dilution). The cells were washed three times with PBS for 5 min each, followed by a 1-h incubation at room temperature with Alexa Fluor 488-conjugated goat anti-rabbit secondary antibodies (Thermo Fisher Scientific, USA). Actin filaments were stained with tetramethylrhodamine B isothiocyanate (TRITC)-conjugated phalloidin (Sigma-Aldrich, USA), and the nuclei were stained with DAPI (Invitrogen, USA). Fluorescent images were captured using an Olympus IX81 inverted research microscope (Olympus, Japan) with appropriate fluorescence filters, and the staining results were analyzed using ImageJ software.

### Cellular fractionation

To examine the expression of YAP and TAZ in the cytoplasm and nucleus, the NE-PER™ Nuclear and Cytoplasmic Extraction Kit (Thermo Fisher Scientific, USA) was used to isolate nuclear and cytoplasmic protein components. The procedure was as follows: 1 × 10^6^ cells were collected and pelleted in a 1.5 ml centrifuge tube. A total of 100 μl of pre-chilled CER I solution was added, followed by vigorous vortexing for 15 s, and the tube was incubated on ice for 10 min. Then, 5.5 μl of pre-chilled CER II solution was added, vortexed for 5 s, incubated on ice for 1 min, vortexed again for 5 s, and centrifuged at 16 000 × g for 5 min. The supernatant, containing the cytoplasmic protein fraction, was transferred to a clean, pre-chilled 1.5 ml centrifuge tube and stored at 4 °C. The remaining pellet was resuspended in 50 μl of pre-chilled NER solution, vortexed vigorously for 15 s, and incubated on ice for 10 min. This step was repeated four times. Finally, the mixture was centrifuged at 16 000 × g for 10 min, and the supernatant, containing the nuclear protein fraction, was transferred to a clean, pre-chilled tube and stored at 4 °C. For long-term storage, both the cytoplasmic and nuclear fractions were kept at −80 °C. Western blot analysis was subsequently performed to quantify the levels of YAP and TAZ in the cytoplasmic and nuclear compartments.

### Wound healing assay

Cells from each group were seeded into 6-well plates at an appropriate density (approximately 5 × 10^5^ cells per well). Once the cells reached 80%–90% confluence, the wound healing test was initiated. A sterile 200 μl pipette tip was used to vertically scratch the cell monolayer, creating a straight line. At least three scratches were made per well to ensure result reproducibility. The cells were gently washed twice with PBS to remove floating cells generated by the scratching process. Fresh medium containing 10% fetal bovine serum (Gibco, USA) was then added, and the cells were cultured further. The closure of the wounds was observed at 0, 24, and 48 h using an inverted microscope (Olympus, Japan), with images captured at the same position for each time point. The width of the wound area at each time point was measured and calculated using ImageJ software (NIH, USA) to compare the wound closure rates between different treatment groups.

### MTT cell proliferation assay

Cells were seeded into 96-well plates at a density of approximately 1 × 10^4^ cells per well, with three replicates for each group. After 24 h of incubation, treatments were initiated by adding appropriate concentrations of Quercetin, CA-NPs, and CA-NPs-Quercetin to the respective groups, while the control group received an equal volume of culture medium. The cells were incubated for 24, 48, and 72 h. Four hours before the end of each time point, 10 μl of MTT solution (5 mg/ml, Sigma-Aldrich, USA) was added to each well, followed by incubation for another 4 h. After incubation, the medium was carefully removed, and 150 μl of DMSO (Sigma-Aldrich, USA) was added to each well to dissolve the purple formazan crystals. Absorbance was measured at 570 nm using a microplate reader (Bio-Rad, USA). Cell viability was calculated based on the absorbance values, with the control group set as 100% viability. The survival rates of the different treatment groups were compared at 24, 48, and 72 h.

### Cell colony formation assay

Cells were seeded into 6-well plates at a density of 3 × 10^3^ cells per well. After 24 h of incubation, Quercetin, CA-NPs, and CA-NPs-Quercetin were added to the respective groups, and the cells were incubated for an additional 24 h. The cells were then washed with PBS, replaced with a complete DMEM medium, and cultured for another 15 days. After the incubation period, the cells were fixed with 75% ethanol and left overnight at 4 °C. Following fixation, the cells were stained with crystal violet for 30 min, after which any unbound dye was gently rinsed off. The stained colonies were then observed for analysis.

### Transwell migration assay

To evaluate the migration ability of mouse airway smooth muscle cells, a Transwell migration assay was performed. An 8 μm pore-sized Transwell insert (Corning, USA) was placed into a 24-well plate. The airway smooth muscle cells were resuspended in serum-free DMEM/F12 medium (Gibco, USA) and seeded into the upper chamber of the Transwell insert at a density of 2 × 10^4^ cells per well. The lower chamber was filled with DMEM/F12 medium containing 10% fetal bovine serum. The plate was then incubated at 37 °C in a 5% CO_2_ incubator for 24 h. After incubation, the non-migrated cells in the upper chamber were gently removed with a cotton swab. The cells that had migrated to the lower surface were fixed with 4% paraformaldehyde and stained with crystal violet (Sigma-Aldrich, USA). The number of migrated cells was counted under a microscope (Nikon, Japan) to assess the migration capacity of the cells in each group.

### Histopathological examination

Histopathological examinations were performed on mice injected with nanoparticles via the tail vein. Blood and major organs (e.g., liver, kidneys, and lungs) were collected at regular intervals. Ten healthy adult mice (C57BL/6, 6–8 weeks old, Experimental Animal Center, China) were used for the study. The nanoparticles were administered at a dose of 10 mg/kg body weight, and major organs were collected on days 7, 14, and 28 after injection. Following anesthesia, the mice were perfused with PBS (calcium- and magnesium-free, Gibco, USA) followed by 10% neutral formalin (Sigma-Aldrich, USA) via cardiac perfusion. The organs were then harvested and fixed in 10% neutral formalin for 24 h. After fixation, the tissues were dehydrated, cleared, and embedded in paraffin. Tissue sections with a thickness of 5 μm were cut using a microtome (Leica, Germany) and mounted on slides for further analysis. After baking the tissue sections, deparaffinization and rehydration were performed using xylene and 95% ethanol. The sections were then washed four times with PBS and underwent antigen retrieval using boiling 1× EDTA. Endogenous peroxidase activity was blocked with an endogenous peroxidase-blocking reagent. Following this, primary antibodies were added, and the sections were incubated overnight at 4 °C. After washing with PBS, a reaction enhancer was applied, and the sections were incubated for 30 min. Subsequently, HRP-labeled anti-mouse/rabbit IgG polymers with high sensitivity were added, followed by a 30-min incubation with the chromogenic substrate. Hematoxylin was used for counterstaining. After rinsing with 1× PBS for blue color correction, the sections were dehydrated and mounted. Finally, the pathological changes in the tissue sections were observed using an optical microscope (Olympus, Japan), and images were recorded. Special attention was given to inflammation, apoptosis, and tissue damage. These steps systematically assessed the histopathological changes induced by different groups of nanoparticles in mice, comparing the effects on major organs such as the liver, kidneys, and lungs across different treatment groups.

### MASSON staining

MASSON staining was performed using a MASSON staining kit (C0189S, Biotem Technology Co., Ltd.) on lung tissue sections to observe collagen deposition. Hematoxylin, acidic fuchsine, and light green were mixed in proportion to prepare the mixed solution. The lung tissue sections were stained with the mixed solution, followed by differentiation with acidic alcohol or acidic water solution to make cell nuclei and other structures clearly visible. The sections were counterstained with methyl blue or phenol blue to stain collagen fibers. Dehydration, transparency, and mounting followed the standard histology procedures. The stained area of each paraffin-embedded section (blue) was outlined using an Axiovert 200 M optical microscope (Carl Zeiss, Jena, Germany), and then Image-Pro Plus software (Version X; Adobe, San Jose, CA) was used to quantify the area occupied by collagen fibers (blue) as a percentage of the total examined area.^32^

### Biochemical analysis

To assess the systemic toxicity of nanoparticles, serum biochemical parameters were measured by collecting blood from mice and using an automated biochemical analyzer (Hitachi, Japan) to evaluate levels of alanine aminotransferase (ALT), aspartate aminotransferase (AST), blood urea nitrogen (BUN), and creatinine (Cr). Healthy adult mice (C57BL/6, 6–8 weeks old, Experimental Animal Center, China) were randomly divided into two groups: a control group and the CA-NPs-Quercetin group, with 10 mice in each group. The mice were injected with nanoparticles via the tail vein at a dose of 10 mg/kg. After 28 days, blood samples were collected from the retro-orbital venous plexus using a sterile needle, and serum was separated by centrifugation at 3000 × g for 10 min. The collected serum samples were then analyzed using the automated biochemical analyzer to measure the levels of ALT, AST, BUN, and Cr. Changes in these biochemical markers were used to evaluate the effects of nanoparticles on liver and kidney function at different time points, providing essential data for determining the systemic toxicity and safety profile of the nanoparticles.

### Establishment of the asthma mouse model

In this study, male BALB/c mice aged 6–8 weeks and weighing 18–22 g (purchased from Charles River Laboratories) were used. All mice were housed in a temperature-controlled environment (22 ± 2 °C) with a humidity of 50 ± 10% under a 12-h light/12-h dark cycle. The mice had free access to standard laboratory feed and drinking water. All experiments were reviewed and approved by the Animal Ethics Committee and were conducted in accordance with relevant animal care guidelines.

The asthma model was established through ovalbumin (OVA) sensitization and challenge as follows:^33^ (1) Sensitization phase: On days 0 and 14, mice were sensitized by intraperitoneal injections of 10 μg OVA (Sigma-Aldrich, USA) mixed with 2 mg aluminum hydroxide (Thermo Fisher Scientific, USA) in a total volume of 200 μl using PBS as the solvent; (2) Challenge phase: From days 21 to 27, the mice were exposed daily to aerosolized 1% OVA solution (dissolved in saline) for 30 min to induce OVA challenge. This protocol successfully established the asthma model in the mice.

### Drug treatment

Drug treatment began on day 21, concurrent with the OVA aerosol challenge, and continued for 4 weeks, with injections administered three times per week until day 48. The treatment groups were as follows: the control group received tail vein injections of an equivalent volume of saline; the empty nanoparticle group received 20 mg/kg of unloaded CA-NPs; the Quercetin group received 20 mg/kg of free Quercetin; and the CA-NPs-Quercetin group received 20 mg/kg of CA-NPs-Quercetin. All treatments were administered three times per week for 4 weeks to evaluate the therapeutic effects of each treatment in the asthma model.

### Isolation and culture of primary airway smooth muscle cells from mice

Airway tissues were harvested from anesthetized mice and rinsed with ice-cold PBS (Gibco, USA) to remove blood and impurities. The tissues were then cut into small pieces, approximately 1 mm^3^, and placed in a digestion solution containing 1 mg/ml collagenase II (Gibco, USA) and 0.5 mg/ml trypsin (Sigma-Aldrich, USA). The tissues were incubated at 37 °C for 1 h with gentle agitation every 15 min. After digestion, the cell suspension was filtered through a 70 μm cell strainer (BD Falcon, USA) to obtain single cells. The collected cell suspension was centrifuged at 300 × g for 5 min, the supernatant was discarded, and the cells were resuspended in DMEM/F12 medium (Gibco, USA) containing 10% fetal bovine serum (Gibco, USA) and 1% penicillin–streptomycin (Gibco, USA). The resuspended cells were then seeded into culture flasks and incubated at 37 °C in a 5% CO_2_ incubator (Thermo Fisher Scientific, USA). Once the cells reached 80% confluence, they were passaged for further experiments.

### AHR and lung function measurement

Airway reactivity was assessed using a dual-chamber plethysmograph (Buxco, Wilmington, NC, USA). Forty-eight hours after the final OVA challenge, mice were placed in the chamber for respiratory monitoring. Noninvasive lung function tests were performed, and the control group, asthma model group, empty nanoparticle group (CA-NPs group), Quercetin group, and CA-NPs-Quercetin group were treated with increasing doses of methacholine (Mch) (0, 10, 25, 50, and 100 mg/ml) (n = 3 per group). The enhanced pause (Penh) values were measured after Mch stimulation. The baseline Penh value was determined using saline treatment (NS), and the percentage increase in Penh at different Mch concentrations (Penh/NS − 1)% was used as an indicator of airway reactivity. Additionally, lung function, including lung compliance and lung resistance, was measured using the FlexiVent system (SCIREQ, Canada). All measurements were conducted on the seventh day following the asthma induction, with each group of mice measured three times, and the average value was taken for analysis. These data systematically evaluated airway reactivity and lung function changes in each group of mice.

### Bronchoalveolar lavage fluid (BALF) collection and analysis

At the end of the experiment, BALF was collected by intubating the trachea of the mice and slowly perfusing 1 ml of sterile saline into the lungs. The BALF was then gently recovered. Total cell counts in the BALF were determined using a hemocytometer (Hausser Scientific, USA), and inflammatory cell types were differentiated and counted using Diff-Quik staining (Thermo Fisher Scientific, USA). The BALF was then centrifuged at 1000 × g for 10 min, and the supernatant was collected. The levels of inflammatory cytokines in the BALF, such as IL-4, IL-10, and IFN-γ, were measured using ELISA kits (eBioscience, USA) to assess the severity of lung inflammation and the effects of different treatments on cytokine levels.

### Histopathological examination

After excising the mouse lung tissue, it was fixed in a 10% neutral buffered formalin solution (Sigma-Aldrich, USA), followed by dehydration in a graded series of ethanol and embedding in paraffin. The tissue sections were cut at a thickness of 4 μm and stained with hematoxylin and eosin (H&E) (Thermo Fisher Scientific, USA) to observe structural changes and inflammatory infiltration in the lung tissue. The stained sections were then examined under a microscope (Nikon, Japan), and images were captured to further assess the impact of different treatments on the lung tissue.

### *In vivo* imaging experiment

After tail vein injection of fluorescently labeled CA-NPs-Quercetin, an *in vivo* imaging system (PerkinElmer, USA) was used to observe the biodistribution of the nanoparticles in mice. Healthy adult mice (C57BL/6, 6–8 weeks old, Experimental Animal Center, China) were randomly assigned to different experimental groups. Following the injection of fluorescently labeled CA-NPs-Quercetin, *in vivo* imaging was performed at various time points (0, 3, 6, 24, 48, and 72 h). Fluorescent signals were recorded at each time point, with a focus on the accumulation and distribution of nanoparticles in major organs such as the liver, kidneys, and lungs. This allowed for the assessment of the biodistribution characteristics and potential targeting properties of the nanoparticles *in vivo*.

### Data analysis

All experimental data were analyzed using GraphPad Prism 9.0 software. The results are expressed as mean ± standard deviation (SD). Group comparisons were performed using one-way analysis of variance (ANOVA) followed by *post hoc* tests. A *p*-value of < 0.05 was considered statistically significant.

## SUPPLEMENTARY MATERIAL

See the supplementary material for Figs. S1–S3.

## Data Availability

The data that support the findings of this study are available from the corresponding authors upon reasonable request.

## NOMENCLATURE

AHR: Airway hyperreactivity
ALT: Alanine aminotransferase
AST: Aspartate aminotransferase
BALF: Bronchoalveolar lavage fluid
BUN: Blood urea nitrogen
CA-NPs: Chitosan-alginate nanoparticles
CA-NPs-Quercetin: Chitosan-alginate nanoparticles loaded with Quercetin
COPD: Chronic obstructive pulmonary disease
Cr: Creatinine
ECIS: Electric cell-substrate impedance sensing
EE: Encapsulation efficiency
FTIR: Fourier transform infrared spectroscopy
H&E: Hematoxylin and eosin
HASMCs: Human airway smooth muscle cells
HPLC: High-performance liquid chromatography
LC: Loading capacity
OVA: Ovalbumin
PBS: Phosphate-buffered saline
SEM: Scanning electron microscopy
TAZ: Transcriptional coactivator with PDZ-binding motif
TEM: Transmission electron microscopy
TGA: Thermogravimetric analysis
UV–Vis: UV–visible spectrophotometry
YAP: Yes-associated protein
